# Case Report: Segmental spinal cord hypoplasia in a Labrador Retriever puppy

**DOI:** 10.3389/fvets.2026.1778369

**Published:** 2026-04-10

**Authors:** A. L. Kamp, A. Martínez Ruiz, T. Karouei, A. R. Tellegen, L. L. Van Stee

**Affiliations:** 1 Division of Diagnostic Imaging, Department of Clinical Sciences, Faculty of Veterinary Medicine, Utrecht University, Utrecht, Netherlands; 2 Division of Veterinary Pathology, Department of Biomolecular Health Sciences, Faculty of Veterinary Medicine, Utrecht University, Utrecht, Netherlands; 3 Division Surgery of Companion Animals, Department of Clinical Sciences, Faculty of Veterinary Medicine, Utrecht University, Utrecht, Netherlands

**Keywords:** canine, congenital, deformity, MRI, myelodysplasia, neurology, segmental spinal dysgenesis

## Abstract

This case report describes the clinical, diagnostic imaging, and histopathologic findings of spinal cord hypoplasia in a 13-week-old, intact male Labrador Retriever. The dog was first presented at 6 weeks of age with chronic and progressive gait abnormalities and fecal and urinary incontinence. On clinical examination, the puppy was bright, alert, and responsive but smaller than its siblings and showed poor muscle mass. No abnormalities were found in the general examination. Neurologic examination revealed mild kyphosis of the lumbar spine, ambulatory spastic paraparesis, proprioceptive pelvic limb ataxia, and a “bunny-hopping” gait. Orthopedic examination did not show any additional abnormalities. Bloodwork for infectious and metabolic etiologies was within normal limits. Magnetic resonance imaging (MRI) of the vertebral column revealed thinning of the thoracolumbar spinal cord with a compensatory increase in subarachnoid space volume, suggestive of spinal cord hypoplasia. In consultation with the owner, euthanasia was elected, given the poor expected quality of life. Postmortem examination showed moderate lumbar kyphosis and mild thoracolumbar scoliosis. Macroscopically, the thoracolumbar spinal cord was multifocally thinned with a markedly irregular dorsolateral surface. Histological examination demonstrated markedly thinned segments with bilateral and symmetric dorsolateral white and grey matter hypoplasia, with the remnant white matter displaying an abnormal conformation with prominent edematous changes. Segmental spinal cord hypoplasia is described as a rare disorder in humans and a few animal species. To the authors’ knowledge, this is the first reported case of segmental spinal cord hypoplasia in a dog.

## Introduction

Generally, the abnormal spinal cord development is referred to as spinal myelodysplasia (1–3). Due to their close embryonic origin, a wide range of vertebral malformations have been associated with spinal cord anomalies. In those cases, the terminology “spinal dysraphism” is often used (2, 4–6). The most frequently reported vertebral abnormalities in combination with spinal cord hypoplasia in ruminants include the complete absence or fusion of vertebral segments, narrowing of the vertebral canal due to anomalies of vertebral bodies or articular processes, or other mild defects such as kyphoscoliosis (4).

The term “spinal dysraphism” encompasses a heterogeneous group of dorsal midline defects affecting the skin, vertebrae, and spinal cord, arising from abnormal development of the ectoderm, mesoderm, and neurectoderm, and includes malformations such as spina bifida, meningocele, meningomyelocele, syringomyelia, and split cord anomalies (diastematomyelia) (3, 7). In both human and veterinary medicine, spinal dysraphism is clinically classified as open or closed based on whether neural tissue is exposed or covered by intact skin (1, 2). Open spinal dysraphism is characterized by herniation or exposure of nervous tissue through defects in the meninges and/or vertebral arches and is typically identified during the neonatal period. In contrast, closed spinal dysraphism (CSD), also referred to as spina bifida occulta, consists of lesions covered by skin and may present with or without associated soft tissue abnormalities (2). CSD is periodically observed in dogs and cats, and multiple anomalies may coexist in affected individuals (8–10).

In human medicine, segmental spinal cord hypoplasia is associated with segmental spinal dysgenesis (SDD), and only 22 human case reports have been published to date (11). SDD in humans is defined by focal agenesis or dysgenesis of the thoracolumbar or lumbar vertebrae and the corresponding segment of the spinal cord. The cervical spinal cord is typically unaffected, whereas the affected segment is thinned or absent, with a bulky, thickened cord positioned distally to the affected segment. This anomaly is frequently associated with kyphoscoliotic deformities and several vertebral malformations and is attributed to abnormal notochord development during early embryogenesis (11–13).

To the authors’ knowledge, only a few bovine cases (4, 14–16), two cases in domesticated cats (17, 18), and one case in a wild cat (19) have been published to date, with all cases showing varying degrees of spinal myelodysplastic comorbidities with or without vertebral involvement.

This case report describes the clinical presentation, diagnostic imaging, and histopathology of spinal myelodysplasia in a 13-week-old intact male Labrador Retriever, characterized by thoracolumbar spinal cord hypoplasia without gross skeletal abnormalities. Changes are most consistent with earlier publications describing segmental spinal cord hypoplasia.

## Case description

### Clinical presentation

A 6-week-old intact male Labrador Retriever was presented to the referral clinic with the clinical complaint of an abnormal gait of the hind limbs in combination with fecal and urinary incontinence. The puppy was smaller than its littermates. Clinical signs had been present for at least the last 2–4 weeks. No other litter mates showed clinical signs, although one died on the first night after birth due to an unknown cause, and one was stillborn. A healthy female littermate was also brought to the clinic for comparison. Videos of the dog at 1, 3, 4, and 13 weeks old are attached as Supplementary Videos S1–S4.

At initial presentation, the general examination showed that the puppy was smaller than his female littermate but was otherwise unremarkable. On neurological examination, a slight spinal kyphosis in the lumbar region was noted. Ambulatory spastic paraparesis and proprioceptive pelvic limb ataxia were suspected, although a complete evaluation was hampered due to the patient’s age. Only slight spinal reflexes could be induced in both this puppy and its littermate. Perineal and anal reflexes were present. Urinary and fecal incontinence were suspected due to continued urinary dribbling and loss of fecal droplets. Orthopedic examination did not reveal any additional abnormalities. Because the owners had noticed some improvement prior to this first visit, a conservative approach was instituted, and a re-check appointment was scheduled 2 weeks later.

At the re-check appointment, at 8 weeks of age, the patient was clearly fecal- and urinary-incontinent, with obvious involuntary loss of urine and fecal material while walking, and showed persistent and more obvious paraparesis and hind limb ataxia. At a faster pace, the patient began bunny hopping. There was kyphosis visible at the level of the lumbar spinal column; palpation and passive movements did not show any abnormalities or pain. Postural reaction testing demonstrated delayed responses in the right hind limb. All spinal reflexes and muscle tone were normal, although there was suspicion of a mild hyperreflexia response due to some clonus in the right hind limb after percussion of the right patellar reflex. The remaining neurological examination was within normal limits. Based on the neurological examination, neuroanatomic localization was expected within the thoracolumbar (T3–L3) and lumbosacral spine (L4–S3), and the patient was scheduled for blood analysis and diagnostic imaging with magnetic resonance imaging (MRI).

At 10 weeks of age, the patient returned for further workup, and no additional clinical signs had emerged. Serology for *Neospora caninum* (FASTest *Neospora caninum* rapid immunochromatographic test with additional MegaFLUO® *Neospora caninum* for the indirect semiquantitative immunofluorescence detection of specific IgG antibodies in case of a positive FASTest) and *Toxoplasma gondii* (MAST®ID Rapid Latex Agglutination Test) were negative. Biochemistry showed no abnormalities in potassium, sodium, glucose, creatine kinase, ammonia, creatinine, total protein, and albumin (20). Additional thyroxine (T4; 54 nmol/L, reference 19–46 nmol/L) showed a mild increase, but thyroid-stimulating hormone (TSH; 0.05 μg/L, reference <0.6 μg/L) was unremarkable. Hematology showed a slight decrease in hematocrit (0.31 L/L, reference 0.42–0.61 L/L), consistent with the patient’s age, and was otherwise unremarkable.

The father of the patient had been screened prior to breeding for several congenital neuromuscular diseases and was found to be negative for conditions including myotubular myopathy, exercise-induced collapse (EIC), centromuscular myopathy (CNM), and degenerative myopathy (DM). Because no myopathy was expected, no additional testing was performed.

### Diagnostic imaging

Radiographic projections of the pelvis at 11 weeks of age, including a ventrodorsal view with extended legs, flexed legs (“frog position”), and a distraction view, obtained using the Vezzoni-Modified Badertscher Hip Distension Device, revealed no convincing signs of coxofemoral dysplasia or other abnormalities of the pelvis. No radiographic projections of the vertebral column were acquired.

**Figure 1:**
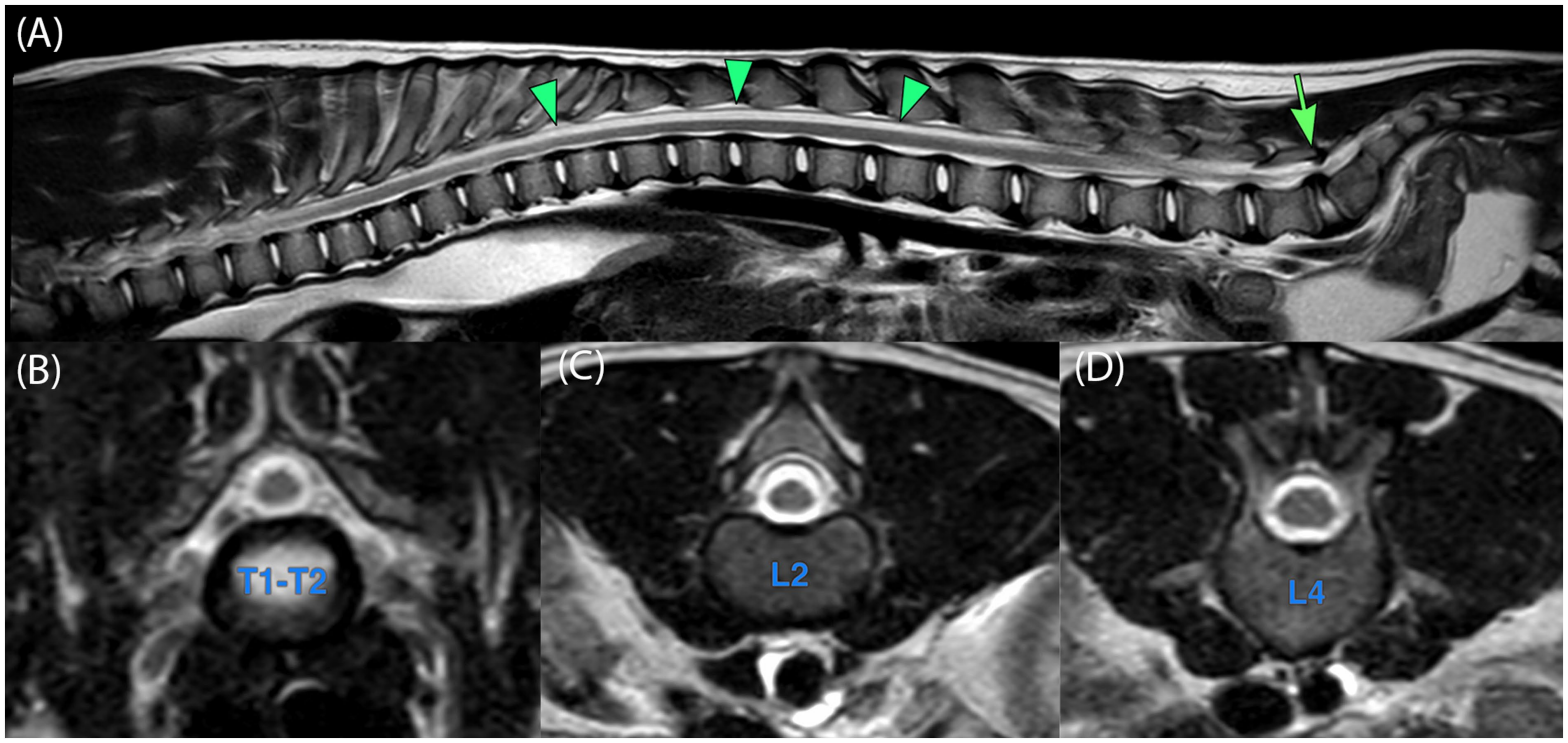
Magnetic resonance imaging (MRI) of the spinal cord. **(A)** Midsagittal T2-weighted image of the entire spinal cord demonstrates the thinned spinal cord (arrowheads), approximately from T2 to L2. The arrow depicts lumbosacral stenosis. **(B–D)** Transverse T2-weighted image of the spine, **(B)** at the level of T1–T2 illustrating the presumably normal spinal cord, **(C)** illustrating the segmentally thin spinal cord at the level of L2, and **(D)** presumably the normal spinal cord caudal to the abnormal segment at the level of L4.

Subsequently, MRI of the brain and entire vertebral column was performed on a 1.5-Tesla scanner (Philips Ingenia 1.5 T MRI, Eindhoven, The Netherlands). There were 7 cervical, 13 thoracic, 7 lumbar, and 3 (not yet fused) sacral vertebrae. The neurocranium was unremarkable. The diameter of the spinal cord was reduced from the T1–T2 intervertebral disc space to the L2–L3 intervertebral space (Figures 1A,B,D). The spinal cord at this level was ventrally positioned within the spinal canal. The subarachnoid cavity, especially dorsally to the spinal cord, showed an increase in volume, occupying the remaining spinal canal. The spinal cord: dorsal subarachnoid space ratio was approximately 50%. The spinal cord was diamond-shaped due to the indentations on the dorsolateral aspects. Multifocal ill-defined T2W hyperintensities were noted within the parenchyma of the thoracolumbar spinal cord. Caudal to L3, the lumbar intumescence had the expected increase in diameter (Figure 1D). From cranial to T2, the spinal cord regained its normal diameter. There was no dilatation of the central canal (Figures 1B–D).

The lumbosacral intervertebral disc was teardrop-shaped and T2W hyperintense. The annulus fibrosus mildly protruded dorsally toward the vertebral canal with partial attenuation of the ventral epidural fat. A subtle step formation of the sacrum and enlargement of the ligamentum flavum/articular processes caused effacement of the epidural fat dorsolaterally to the cauda equina. This resulted in moderate obliteration of epidural fat surrounding the nerve roots of the cauda equina, as seen on the transverse T2W images. The fat signal remained visible between the various nerve tissues.

The imaging findings were suggestive of a congenital thoracolumbar myelopathy, although normal variation for the patient’s young age could not be excluded. The findings of the lumbosacral junction were concerning for lumbosacral stenosis, although these changes could be aggravated by positioning. Mild ascites, likely age-related, and moderate oesophageal fluid distension, likely secondary to anesthesia, were noted as well, although functional esophageal abnormalities could not be excluded.

Due to the MRI results, the lack of any clinical improvement, continued severe fecal and urinary incontinence, and the expected poor quality of life, the owners elected euthanasia at 13 weeks of age, and the patient was submitted for postmortem examination.

### Postmortem examination and histopathology

At necropsy, the body condition was appropriate for the dog’s age, with moderate musculature and scant fat reserves. The vertebral column showed a moderate dorsal curvature of the cranial lumbar region (kyphosis) and a slight lateral deviation of the thoraco-lumbar spine (mild scoliosis).

**Figure 2:**
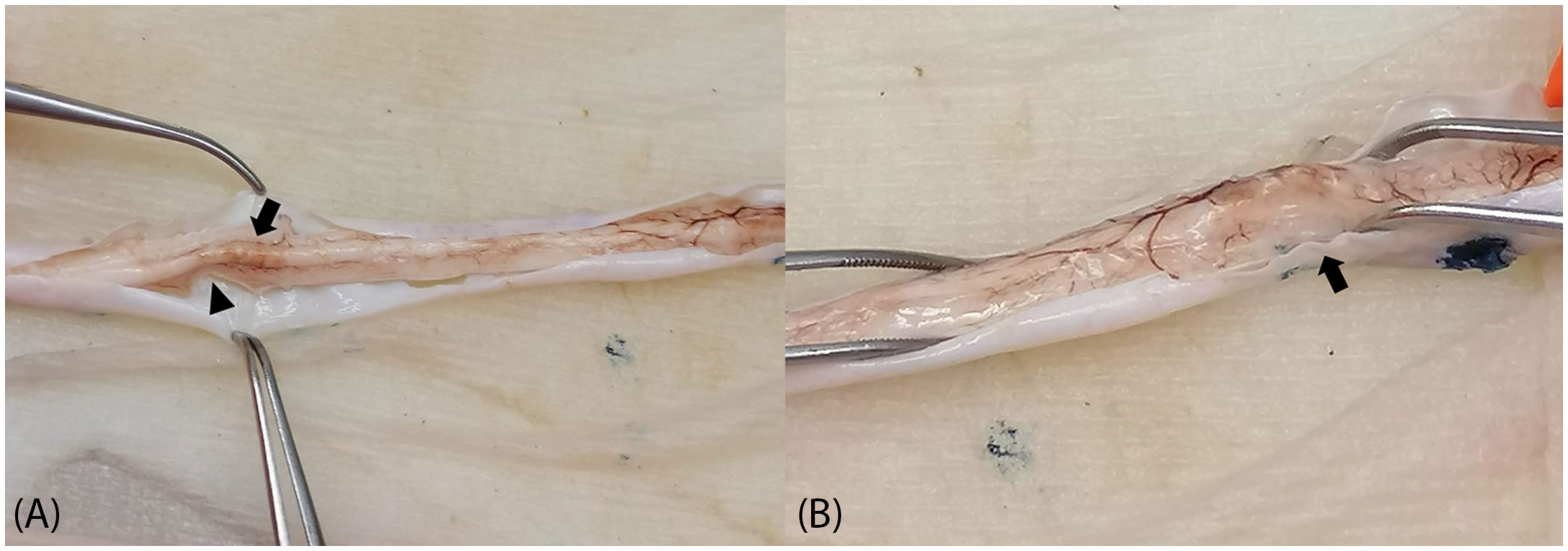
Gross pathology. **(A)** Thoracolumbar spinal cord. Moderately thin spinal cord showing multifocal irregular indentations alongside the dorsolateral aspects. The caudal thoracic cord presents a prominent dorsal projection (arrow) limited by a bilateral longitudinal indentation, and the adjacent parenchyma appears markedly thinned (arrowhead). **(B)** Cranial lumbar spinal cord. Irregular dorsal surface with a focal area of markedly thinned dorsolateral parenchyma (arrow), without the presence of a central dorsal projection as seen at thoracic levels. Note the irregularly oriented and distended subdural vasculature surrounding the areas of markedly indented parenchyma, suggesting abnormal vasculature anatomy and supporting the diagnosis of a congenital malformation.

The spinal cord was covered by uniform, white, and shiny dura mater without macroscopic signs of inflammation, hemorrhage, neoplastic masses, or attachment to the vertebrae, and a clear presence of central canal stenosis was not noted during necropsy. After a week of fixation in buffered formalin, the dura mater was cut along the dorsal aspect of the whole spinal cord, showing a moderately to markedly irregularly thinned parenchyma in the thoracic and lumbar regions, surrounded by a small amount of transparent, clear, and watery cerebrospinal fluid (Figure 2). The dorsal aspect of the cord in these areas was irregular and presented multifocal and longitudinal indentations that reached the cranial levels of the lumbosacral intumescence. On transverse cuts at different thoracolumbar levels, the parenchyma often appeared irregular and mottled, with irregular indentations on the cut surface mainly, but not restricted to the dorsal areas of the parenchyma, measuring up to 1 mm in diameter and frequently coalescing.

Several peripheral nerves were examined and collected (brachial plexi, sciatic nerves, and phrenicoabdominal nerve), as well as the celiac ganglia, none of which showed remarkable macroscopic changes.

Incidental changes in non-nervous tissue were likely consequences of euthanasia and consisted of pulmonary edema and congestion, minimal hydropericardium, and hepatic congestion. The other organs did not show macroscopic abnormalities.

Histological examination with the hematoxylin and eosin (H&E) stain was performed on two transverse cuts of cervical and lumbar spinal cord, thoracic intumescence, and lumbar intumescence; three transverse cuts of thoracic cord; one transverse cut of right and left celiac ganglia; and one longitudinal cut of phrenicoabdominal nerve.

**Figure 3:**
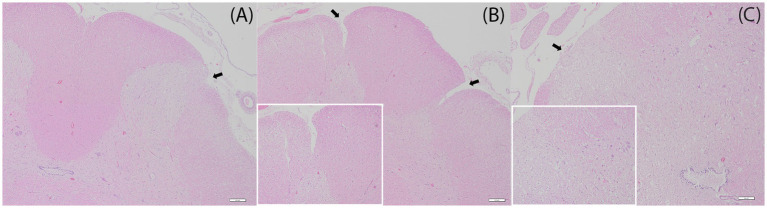
Histology, hematoxylin and eosin. **(A)** Lumbar intumescence, dorsal right, ×4 magnification. The dorsal and dorsolateral white matter is moderately thinned, with markedly decreased parenchyma on the dorsolateral aspects above the dorsal grey matter horn (arrow). Scale bar: 200 μm. **(B)** Lumbar spinal cord, cranial aspects, ×4 magnification. The dorsal white matter presents a prominent papillary zone delimited by two dorsolateral deep clefts (arrows), almost reaching the dorsal horns of the grey matter. Scale bar: 200 μm. (**B** inset) Higher magnification (×10) on the left cleft in the white matter. Note the extensive vacuolation of the neuropil affecting both white and grey matter, more prominent on the left side of the image. **(C)** Lumbar spinal cord, caudal aspects, ×4 magnification. Dorsal and dorsolateral markedly thinned white matter, with a focal area of almost absent white matter (arrow). There is prominent vacuolation in the remnants of white matter and diffusely affects the grey matter (inset). The central canal shows no remarkable changes. Scale bar: 200 μm. (**C** inset) Inset, ×10 magnification.

Coinciding with the areas previously described, the thoracolumbar spinal cord and the cranial areas of the lumbar intumescence showed remarkable histological changes (Figure 3). These segments were markedly thinned to almost half the diameter of the cervical spinal cord. Bilaterally and symmetrically, the dorsolateral white matter and sometimes the dorsal grey matter (only in lumbar regions) are markedly decreased in size, often with a central dorsal papillary projection of white matter limited by two dorsolateral, variably deep indentations reaching up to the grey matter. Laterally to these clefts, there was a remarkable decreased amount of white matter, and these areas often showed a moderate to marked spongy appearance, sometimes also extending into the lumbar dorsal grey matter (consistent with intravital edema). In caudal lumbar areas, the dorsal and dorsolateral white matter was almost completely absent, with marked edematous changes that extended deeper into the grey matter, which also showed decreased numbers of neurons in comparison with the ventral areas and other dorsal areas at more cranial levels. Throughout the whole length of the spinal cord, no morphological changes affecting the central canal were noted. No abnormalities were found in the cervical spinal cord, celiac ganglia, or phrenicoabdominal nerve.

**Figure 4:**
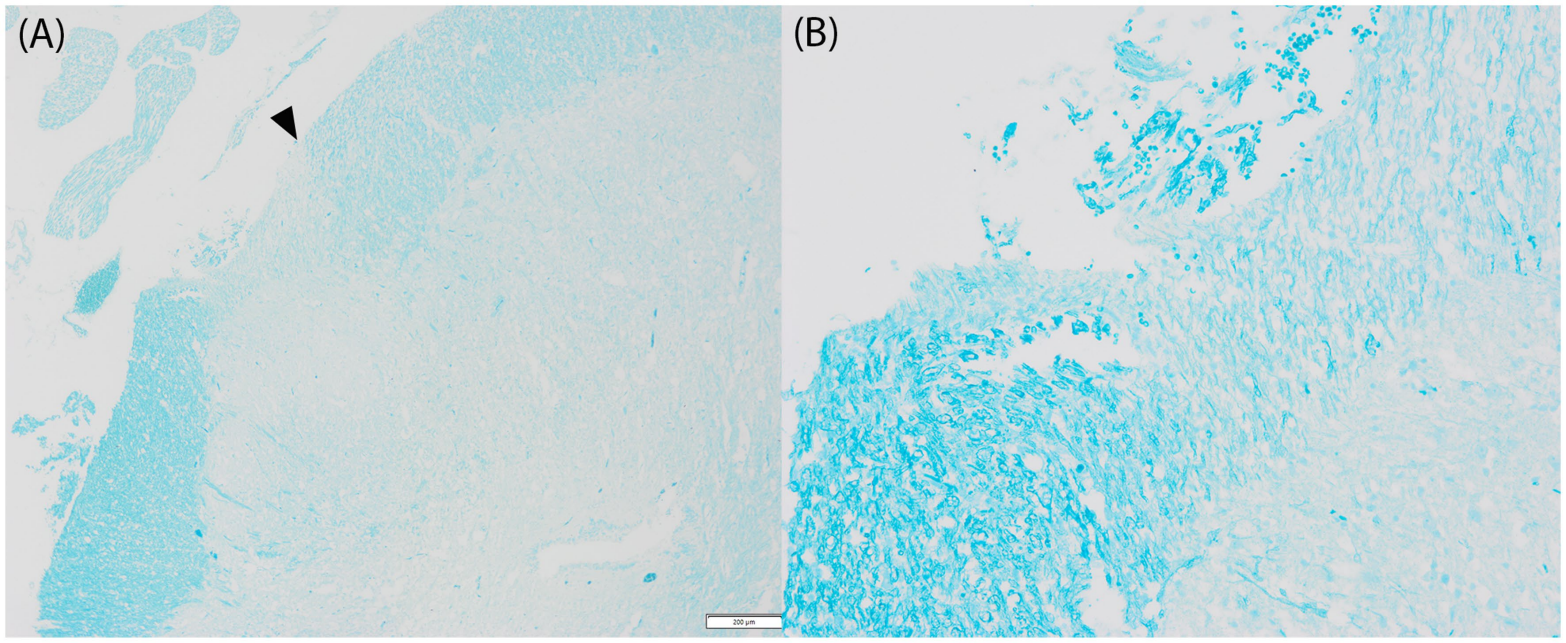
Histology, Luxol Fast Blue. **(A)** Lumbar spinal cord, caudal aspects, ×4 magnification. The remnants of the thinned dorsal white matter (left) present normal myelinization, with an abrupt change in the staining properties at the dorsolateral aspects (left from arrowhead). The few remnants of white matter in this area showed markedly decreased myelin density, with abundant interspersed clear spaces that extend less prominently toward the more lateral white matter (top right, right from arrowhead). Scale bar: 200 μm. **(B)** Inset × 20 magnification.

Both macroscopic and histological changes are compatible with thoracolumbar spinal cord hypoplasia, affecting mainly the dorsolateral white and grey matter (see Figure 4).

## Discussion

This case report describes a 13-week-old intact male Labrador Retriever that presented with progressive and persistent ambulatory spastic paraparesis and proprioceptive pelvic limb ataxia, a bunny hopping gait, and fecal and urinary incontinence. Based on the clinical presentation, diagnostic imaging, and histopathological findings, a suspicion for segmental spinal cord hypoplasia and developmental lumbosacral stenosis was raised.

Segmental spinal cord hypoplasia has been described in a few bovine patients (4, 14–16), two domesticated cats (17, 18), and one wild cat (19). In human medicine, the terminology “segmental spinal cord hypoplasia” is not commonly used; instead, cases are described as a combination of hypoplastic or aplastic features of the spinal cord and dysraphic changes of the vertebral column, collectively known as segmental spinal dysgenesis (SDD) (12, 21). Initially, SSD was described as localized agenesis or dysgenesis affecting the lumbar or thoracolumbar spine, commonly associated with congenital scoliosis and focal abnormalities of the spinal cord and nerve roots (21–24). This definition was later broadened to classify SSD as a form of congenital spinal dysraphism characterized by specific clinical and radiological features. With more recent publications mentioning cases with no clear indication of spinal cord compression and only mild kyphoscoliosis (12, 25) present diagnostic criteria require the presence of: (1) paraparesis or paraplegia, often associated with lower limb anomalies, (2) multisegmented vertebral abnormalities in varying severity, which may result in kyphosis or kyphoscoliosis, (3) absence or malformation of a portion of the spinal cord and corresponding nerve roots at any level from the cervical spine to the sacrum, and (4) preservation of spinal cord tissue distal to the affected segment (11–13, 21, 26, 27).

The presence and severity of vertebral involvement and spinal canal stenosis in combination with segmental spinal hypoplasia classifies cases into either type I or type II SDD (11, 12, 26, 27). Surgical intervention is generally not advised in type I cases (11, 12, 28).

In veterinary medicine, spinal cord malformations remain less well characterized. Myelodysplasia refers to abnormal spinal cord development due to neural tube defects (1, 3, 7, 23), while spinal dysraphism is defined by incomplete closure, a raphe, or defective fusion of the neural tube (29). Segmental spinal cord hypoplasia may be classified as either spinal myelodysplasia or spinal dysraphism based on the extent and presence of vertebral column involvement, although both terms are seen throughout the current literature. Most published ruminant cases on segmental spinal cord hypoplasia show comorbidities with several vertebral abnormalities in differing degrees. Current publications mention vertebral abnormalities as absent (16) to mild (4, 14) to severe (15). The two feline cases showed different abnormal formation of the vertebral column in combination with spinal cord hypoplasia (17, 18). This case showed only very mild changes in the vertebral column with mild kyphosis and scoliosis at the level of the thoracolumbar spine, very mild lumbosacral changes suggestive of lumbosacral stenosis, and no clear central canal stenosis was noted.

In cases of spinal myelodysplasias or spinal dysraphism, clinical signs are typically present at birth or become evident when the neonate first attempts to stand or walk, and they are generally non-progressive. Due to the wide spectrum of myelodysplastic abnormalities, no specific clinical sign reliably differentiates between individual malformations (1, 29). In all published cases of segmental spinal hypoplasia, patients present with a combination of neurological clinical signs, mainly including spasticity (4, 18) or flaccid (15–17, 19) paresis or paralysis of the hind limbs, “inability to stand” (14, 16), and hyperextension of the stifle and tarsal joints (4) with normal to exaggerated reflexes (4) or absent reflexes (16). Also, in b*o*th feline cases, the authors mention pelvic limb paraplegia in combination with urinary and fecal incontinence since birth (17, 18). The patient in this study was presented with ambulatory spastic paraparesis, proprioceptive pelvic limb ataxia, a “bunny-hopping” gait, and urinary and fecal incontinence. This is suggestive of a multisegmental spinal problem. The ambulatory spastic paraparesis and proprioceptive pelvic limb ataxia were most likely caused by segmental thoracic spinal cord hypoplasia (T3-L3 neurolocalization). The “bunny hopping gait” can be seen in some orthopedic diseases, such as hip dysplasia, but it is quite typical in cases of canine myelodysplasias and spinal dysraphism (5). Radiographic evaluation of the hips of the puppy using hip distraction showed no evidence of joint laxity nor anatomical signs of hip dysplasia. Orthopedic examination also did not show any anomalies that could cause gait anomalies, such as joint pain, arthritis, hip laxity, or cruciate disease. Urinary and fecal incontinence, manifested as the constant dribbling of urine, may indicate either a flaccid bladder due to a dysfunction of the hypogastric nerve (L1–L4 neurolocalization), as described in cases of spinal dysplasia, or dysfunction of the pudendal nerve (S1–S3 neurolocalization) (5). In this case, the incontinence may have been associated with lumbosacral stenosis, which, given the age of the dog, would likely be congenital or developmental. In dogs, lumbosacral stenosis is mainly considered a degenerative disease; however, since some dogs with degenerative lumbosacral stenosis are relatively young, there could be a congenital component that causes early degeneration (30). Clinically, SSD in humans most commonly presents with spastic paraparesis and neurogenic bladder, although symptom severity varies depending on the level and extent of malformation, degree of kyphosis, and associated systemic anomalies (12, 28).

Diagnostic imaging of segmental spinal dysgenesis in human medicine typically demonstrates a normally developed proximal spinal cord, followed by a thinned cord segment lacking nerve roots, and a bulky, low-lying distal cord segment (12, 22, 28), very similar to that observed in the present case. In the few reported veterinary studies with diagnostic imaging performed, an “hourglass” appearance of the spinal cord was visible in either myelography (4, 16) or MRI (4). In this case, subtle changes over a relatively long segment were noted, which could be classified as an “hourglass-appearance,” although this was not as clear as in the two previously mentioned articles (Figure 1A).

MRI also revealed multifocal ill-defined T2W hyperintensities within the parenchyma of the thoracolumbar spinal cord. Given the absence of inflammation or increased cellularity, this could indicate irregular areas of intraparenchymal edema, as seen in the histopathology of this case, affecting most of the dorsal and lateral white matter. Comparable to previous publications on segmental spinal hypoplasia, this case presented without macroscopic signs of inflammation, hemorrhage, neoplastic masses, or attachments to the vertebrae. In this case, no central canal stenosis was noted, which was present in one (15) but not all (4, 14, 16) of the other published cases. A clear, irregularly thinned parenchyma on the thoracic and lumbar regions was evident, which is similar to the other published cases (4, 14–19). Comparable to the present case, the histopathology of all cases (4, 14–17, 19) reported clear atrophy in gray and white matter, with evident demyelination of the white matter (4, 14, 16).

In dogs, the most familiar and well-studied spinal cord myelodysplasia is inherited myelodysplasia of Weimaraners (29), with pelvic limb gait disturbances similar to the clinical picture of the present case. In this entity, a known underlying cause is identified: a mutation in exon 2 of the NKX2-8 gene in Weimaraner dogs, which has not been demonstrated in other dog breeds. Affected animals often show additional macroscopic changes (thoracolumbar scoliosis, sternum depression, and abnormal hair streams in the dorsal cervical region). Although the patient did show very mild spinal kyphosis of the lumbar region, no other comparable abnormalities to hereditary Weimaraner myelodysplasia were observed. Additionally, this inherited myelodysplastic syndrome is usually characterized by histological changes affecting the central canal, gray matter, and/or ventral median fissure. White matter hypoplasia (as seen in the present case) is not recognized as a common change in these puppies (1, 3, 29, 31).

To date, the inheritance patterns or the underlying cause of segmental spinal hypoplasia remain undefined (4, 7, 19). Both environmental and biological factors have been implicated in neural tube defects, including genetic predisposition, nutritional imbalance, exposure to drugs or hyperthermia during pregnancy, and maternal infectious or metabolic conditions (32, 33). Notably, in humans, maternal diabetes is associated with a 3- to 5-fold increase in congenital malformations, including neural tube defects such as exencephaly and spina bifida, compared with non-diabetic pregnancies (33). In the present case, no medical abnormalities were present in either parent, and gestation and birth were uncomplicated.

In conclusion, this case describes a 13-week-old intact male Labrador Retriever who was presented with progressive and persistent ambulatory spastic paraparesis, proprioceptive pelvic limb ataxia, a bunny hopping gait, and fecal and urinary incontinence. Clinical examination showed slight spinal kyphosis of the lumbar region but was otherwise unremarkable. No underlying causes were identified. Based on the clinical presentation, diagnostic imaging, and histopathology, a diagnosis of segmental spinal cord hypoplasia was established. Further publications are needed to determine the potential treatment options and the underlying cause in canine patients.

## Data Availability

The original contributions presented in the study are included in the article/Supplementary material, further inquiries can be directed to the corresponding authors.

## Supplementary material

SUPPLEMENTARY VIDEO S1 1 week old.

SUPPLEMENTARY VIDEO S2 3 weeks old.

SUPPLEMENTARY VIDEO S3 4 weeks old.

SUPPLEMENTARY VIDEO S4 13 weeks old.
